# EGFR related mutational status and association to clinical outcome of third-line cetuximab-irinotecan in metastatic colorectal cancer

**DOI:** 10.1186/1471-2407-11-107

**Published:** 2011-03-25

**Authors:** Karen-Lise G Spindler, Niels Pallisgaard, Jan Lindebjerg, Sanne K Frifeldt, Anders Jakobsen

**Affiliations:** 1 Danish Colorectal Cancer Group South, Department of Oncology, Vejle Hospital, Kabbeltoft 25, Vejle, (7100), Denmark; 2 Danish Colorectal Cancer Group South, Department of Biochemistry, Vejle Hospital, Kabbeltoft 25, Vejle, (7100), Denmark; 3 Danish Colorectal Cancer Group South, Department of Pathology, Vejle Hospital, Kabbeltoft 25, Vejle, (7100), Denmark

**Keywords:** metastatic colorectal cancer, KRAS, BRAF, PIK3CA/PTEN mutations, Cetuximab and Irinotecan

## Abstract

**Background:**

As supplement to *KRAS *mutational analysis*, BRAF and PIK3CA *mutations as well as expression of PTEN may account for additional non-responders to anti-EGFR-MoAbs treatment. The aim of the present study was to investigate the utility as biomarkers of these mutations in a uniform cohort of patients with metastatic colorectal cancer treated with third-line cetuximab/irinotecan.

**Methods:**

One-hundred-and-seven patients were prospectively included in the study. Mutational analyses of *KRAS, BRAF *and *PIK3CA *were performed on DNA from confirmed malignant tissue using commercially available kits. Loss of PTEN and EGFR was assessed by immunohistochemistry.

**Results:**

DNA was available in 94 patients. The frequency of KRAS, *BRAF *and *PIK3CA *mutations were 44%, 3% and 14%, respectively. All were non-responders. EGF receptor status by IHC and loss of PTEN failed to show any clinical importance. *KRAS *and *BRAF *were mutually exclusive. Supplementing *KRAS *analysis with *BRAF *and *PIK3CA *indentified additional 11% of non-responders. Patient with any mutation had a high risk of early progression, whereas triple-negative status implied a response rate (RR) of 41% (p < 0.001), a disease control (DC) rate of 73% (p < 001), and a significantly higher PFS of 7.7(5.1-8.6 95%CI) versus 2.3 months (2.1-3.695%CI) (p < 0.000).

**Conclusion:**

Triple-negative status implied a clear benefit from treatment, and we suggest that patient selection for third-line combination therapy with cetuximab/irinotecan could be based on triple mutational testing.

## Background

Selection of patients for epidermal growth factor receptor targeted monoclonal antibodies (MoAbs) based on tumour *KRAS *analysis is a major step towards tailored treatment in metastatic colorectal cancer. An increasing amount of data has demonstrated that response to anti-EGFR MoAbs is confined to patients with *KRAS *wild type tumour [1-4]. Patients harbouring *KRAS *mutations are not likely to benefit from these drugs and *KRAS *testing is now recommended in this setting [5]. Still, a major fraction of the *KRAS *wild-type patients are non-responders, and may not benefit from treatment. The anti-EGFR MoAbs imply a substantial degree of toxicity, and investigations of supplementary predictive factors are therefore highly relevant.

Downstream signalling by the RAS-RAF-MAPK pathway is normally very tightly regulated. However, mutations in the *KRAS *gene render the KRAS protein constitutively active regardless of extracellular EGFR inhibition[2]. Mutations in the genes coding for the other members of the RAS-RAF-MAPK pathway have been identified and may theoretically determine primary resistance to EGFR inhibition in a similar matter[4,6]. This has been suggested by cell line studies and clinical data presented by Nicolantonio et al who investigated *BRAF *and *KRAS *status in 113 patients treated with panitumumab or cetuximab[4,7,7]. Oncogenic activation of BRAF is present in approximately 8-10% of colorectal tumours and seems mutually exclusive from *KRAS *mutations according to the literature. A hotspot in colon 600 accounts for the majority of *BRAF *mutations. However, the relatively low frequency of *BRAS *mutations detected may be due to poor sensitivity assays (e.g direct sequencing) similar to previously published data on *KRAS *analysis[8]. Consequently, methodological aspects warrant investigation, including development of high-sensitive methods.

Activation of the phosphatidylinositol 3-kinase (PI3K/AKT) pathway is another central mechanism of tumour cell regulation. The PI3K/AKT pathway is critical for development of malignant tumours, constitutes a relevant target for anticancer therapy and regulates the central mTOR pathway, which is influenced by chemotherapeutics and new biological agents[6,9,10]. PI3K stimulates the phosphorylation of AKT by interaction with phosphatidylinositol-3-phosphate in the cell membrane. A catalytic subunit of the PI3K is encoded by the *PIK3CA *gene, which harbour activating mutations in 10-30% of colorectal tumours according to the literature[6,11-13]. The PI3K/AKT pathway promotes cellular proliferation, invasion, cell survival and neo-angiogenesis[10,14], and PI3K-initiated signalling is normally inhibited by the phosphatase and tensin homolog (PTEN) gene located on chromosome ten. Consequently, *PIK3CA *mutations and/or loss of PTEN may render malignant cells resistant to EGFR inhibition as described in preclinical studies[15]. This has also been suggested in a recently published clinical trial including 110 patients with metastatic colorectal cancer. A clear association was demonstrated between loss of PTEN/*PIK3CA *mutations and response to different regimes of EGFR-tageted MoAbs. Furthermore, an independent prognostic value regarding progression free survival was reported by multivariate analysis[13,16].

The EGF receptor expression by IHC has been discharged as predictive marker for response, but the previous data has assessed the receptor status in full populations and not in relation to the mutational status. Just recently a small study has suggested a reconsideration of this aspect by demonstrating a positive predictive value of adding the EGFR expression to *KRAS *mutational status. The study included 95 patients treated with FOLFIRI or FOLFOX+ cetuximab treatment, and EGFR expression was investigated by IHC and gene expression on primary tumour tissue [17]. The relevance of performing EGF receptor analysis in third-line treatment for KRAS wild-type disease remains to be determined.

As supplement to *KRAS *analysis, *BRAF *and *PIK3CA *mutations as well as loss of PTEN and EGFR expression may account for additional non-responders to EGFR targeted MoAbs. The few previously published studies have combined data from patients treated with different EGFR targeted antibodies as mono- or combination-therapy in first, second and third-line settings. Various methods for mutational testing have been used. The aim of the present study was to investigate the clinical value of triple *KRAS*, *BRAF *and *PIK3CA *mutational testing by commercially available kits combined with immunohistochemical testing of EGFR and PTEN loss in a material of patients with metastatic colorectal cancer. All patients were treated in a single institution with uniform combination therapy cetuximab-irinotecan (CETIRI).

## Methods

### Patient material

We included 107 heavily pre-treated patients with metastatic colorectal cancer, previously exposed to fluoropyrimidine, irinotecan and oxaliplatin containing regimes and admitted for third line treatment with cetuximab and irinotecan. Clinical eligibility criteria were; Histologically confirmed mCRC (adenocarcinomas) refractory to prior chemotherapy, age over 18 years, ECOG performance status ≤ 2, adequate organ function, and measurable disease according to the Response Evaluation Criteria in Solid Tumours (RECIST)[18]. Patients were treated with a combination of irinotecan (350 mg/m^2 ^q3w) and cetuximab (400 mg/m^2 ^loading dose followed by weekly 250 mg/m^2^). Response was classified according to RECIST. Treatment was terminated upon progression or in 6 patients after prolonged duration of treatment. Paraffin-embedded primary tumours and/or corresponding metastasis (in one patient only metastatic tissue was available and therefore used for testing) were collected from referring departments of pathological. The study was conducted in accordance with the Danish law after approval by the Regional Ethics Committee. Oral and written consent was obtained from all patients.

### DNA purification and KRAS, BRAF and PIK3CA mutational analysis

DNA was extracted from formalin-fixed paraffin-embedded tissue using QIAamp DNA Mini Kit (QIAGEN) after histological confirmation of viable tumour cells on HE stained slides. Mutant *KRAS*, *BRAF *and *PIK3CA *were determined by pre-developed kits identifying seven somatic *KRAS *mutations (G12A, G12R, G12D, G12C, G12S, G12V and G13D), one *BRAF *mutation (E600V) and 4 *PIK3CA *mutations (E542K, E545K, E545D and H1047R) (DxS Ltd, Manchester, United Kingdom). Allele-specific real-time quantitative PCR (qPCR) was performed on an ABI7900HT Sequence Detection System (Applied Biosystems, Foster City, CA) according to manufacturer's recommendation.

### PTEN and EGFR immunohistochemistry

Immunohistochmical stainings were performed on 4 μm thick sections. Briefly, after deparaffination in graded alcohol solutions antigen retrieval was performed by microwaving in Tris-EGTA buffer, pH 9,0. Subsequently, the slides were incubated with antibody against PTEN (Dako, clone 6H2.1, dilution 1:400), and EGFR primary mouse anti-EGFR Mab (EGFR clone H-11, dilution 1:75). The reactions were visualized with Super Sensitive (BioGenex) and ENVISION (DAKO Cytomation-DK) with DAB, followed by counterstaining with haematoxylin. Staining was performed manually.

The PTEN reaction was considered negative if more than 50% of the tumor cells were negative or weakly stained, compared to the adjacent stromal cells which served as internal control.

EGFR positivity was defined according to DAKO guidelines, any membrane staining above background level was considered positive. Tumours were graded with regard to intensity and amount of membrane staining. A score of staining intensity was assigned as follows: 1+ = weak, 2+ = moderate and 3+ = strong membrane staining. The tumour was defined positive if ≥1% of the cells had membranous staining for EGFR according to the DAKO guidelines. A tumour with less than 1% positive cells was considered EGFR-negative. The score was defined according to the percentage of positively stained tumour cells as follows: 0 = less than 1%, 1 = 1-10%, 2 = 10-25%, 3 = 25-50%, 4 = > 50%.

Evaluation was independently performed by two investigators (KGS and JL/SKF). The inter- and intra observer reproducibility of assessments of the IHC staining was tested by calculating Cohen's kappa and showed excellent agreement (data not shown).

### Statistics

The association between mutational status and objective response rates, baseline characteristics and skin-toxicity rates was determined by two-tailed Fisher`s exact test. Patients who ended treatment before the first objective tumour evaluating were included in analysis as non-responders, except from patients experiencing anaphylactic reaction during the first treatment dose. Survival analyses were performed according to the Kaplan-Meier method and survival curves compared by the log-rank test. Progression free survival was defined as time from start of treatment until documented tumour progression or death. Overall survival was calculated from date of first treatment until death of any course. All-p-values were two-sided and considered significant if p ≤ 0.05. Statistics were carried out using the NCSS statistical software 2007 v.07.1.5 (NCSS Statistical Software, Utah 84037, USA, http://www.ncss.com).

## Results

One-hundred and seven patients were prospectively included in the study, and 103 patients completed at least one cycle of combination therapy. The median number of cycles was 6 (range 1-18). Eighty-six patients were evaluable for response according to RECIST. Twenty-one patients received less than 3 cycles and were consequently not evaluated by radiological examination according to study schedule. Study treatment was terminated in three cases because of grade 3-4 allergic reactions during the first infusion. One patient withdrew consent after the first dose for psychological reasons. Furthermore 8 patients showed clinical progression and stopped treatment after one cycle and 10 after the second cycle of treatment. Additional patient characteristics are presented in table 1.

**Table 1:** Patient characteristics

Characteristic	Patients (n = 107)	
	No.	(%)
Age, Years		
Median	62	
Range	38-82	

Gender		
Female	49	(46)
Male	58	(54)

ECOG performance status		
0	55	(51)
1	42	(39)
2	10	(10)

No. of prior chemotherapy regimes for metastatic disease		
2	79	(74)
3	27	(25)
NA	1	(1)

Primary surgery for CRC		
Yes	97	(91)
No	9	(8)
NA	1	(1)

Pre-operative chemoradiation		
Yes	17	(16)
No	88	(82)
NA	2	(2)

Anatomic site		
Colon	40	(37)
Rectosignoideum	36	(34)
Rectum	39	(36)
NA	3	(3)

No. of metastatic sites		
1-2	48	(45)
3-5	57	(53)
NA	1	(1)

No of cycles		
Median	6	
Range	1-18	

Best response		
PR	20	(19)
SD	35	(33)
PD	31	(29)
Clinical progression*	17	(16)
NA	4	(4)

Worst Toxicity/Rash grade		
0	15	(14)
1	41	(39)
2	19	(18)
3	11	(13)
NA	25	(23)

Legend; NA (not assessed), PR, partial response; SD, stable disease, PD progressive disease, * clinical progression (at investigators discretion) in patients who were treated with less than 3 series and consequently not evaluated according to RECIST.

The overall response rate (RR) was 19% (20/103) and 34% (35/103) achieved stable disease. Eighty-three patients (81%) were non-responders as defined by progressive disease (PD) + stable disease (SD) and included 17 patients who showed progression within the first 3 cycles. Skin toxicity data were available in 84% of the patients and graded according to CTC criteria. At the time of analysis 10 patients were still alive, including 3 patients with stable disease. The median observation time was 7.7 months (range 0.8-31). The median progression free survival of the 103 patients who completed the first cycle was 3.9 months (2.6-4.7 95% CI) and the median overall survival 7.3 months (5.8-9.9 95%CI).

### EGFR expression

Sixty-eight tumours were available for EGFR staining. Forty-seven percent were negative, whereas 36 (53%) tumours showed a positive staining including 17 with intensity 2 or greater. EGFR IHC status was not associated with clinicopathological parameters, response or survival in the full cohort (table 2) or in selective analysis in the KRAS wild type patients (data not shown).

**Table 2:** Outcome according to marker status

	Response	Disease	Median PFS		Median OS	
	**Rate**	**Control Rate**	**months (95% CI)**		**months (95% CI)**	

Total (n = 94)	20%	56%	4.2	(2.8-5.1)	8.6	(5.9-10.4)
*KRAS*						
Mutation (n = 41)	0%	45%	2.8	(2.2-4.1)	6.5	(4.8-9.8)
wild type (n = 53)	37%	65%	6.5	(3.5-8.4)	9,9	(6.0-12.2)
	p < 0.000	p > 0.05	p = 0.0007		p > 0.05	
*BRAF*						
Mutation (n = 3)	O%	33%	2.1	(1.9-8.5)	4.5	(3.5-25.7)
Wild type (n = 90)	20%	56%	4.2	(2.8-5.1)	8.6	(5.9-10.4)
	p > 0.05	p > 0.05	p > 0.05		p > 0.05	
*PIK3CA*						
Mutation (n = 13)	0%	15%	2.2	(2.1-2.3)	3.5	(3.0-4.7)
Wild type (n = 81)	23%	63%	4.6	(3.5-6.2)	9.2	(6.6-11.1)
	p = 0.053	p = 0.001	p = 0.0003		p = 0.003	
PTEN IHC						
Loss of PTEN (n = 13)	8%	46%	3.3	(2.1-6.3)	5.9	(3.6-10.9)
Normal expression (n = 59)	23%	57%	4.4	(2.8-6.2)	9.2	(6.9-11.1)
	p > 0.05	p > 0.05	p > 0.05		p > 0.05	
EGFR IHC						
Positive staining (n = 36)	25%	61%	4.3	(2.2-5.7)	9.0	(6.1 -10.9)
Negative staining (n = 32)	22%	43%	2.8	(2.1-6.9)	8.6	(3.5 - 11.1)
	p > 0.05	p > 0.05	p > 0.05		p > 0.05	
Triple mutation						
*mutation (n = 49)	0%	40%	2.3	(2.1-3.6)	5.8	(4.5-9.2)
Negative (n = 45)	41%	73%	7.7	(5.1-8.6)	10.2	(7.1-12.5)
	p < 0.0000	p = 0.001	p < 0.000		p > 0.05	

*Any of the three mutations detected in primary tumour or metastatic tissue.

# In a few cases DNA was not available for testing of all three mutations.

Response and disease control rates calculated by chi-square test.

Progression free survival (PFS) and overall survival (OS) calculated by log-rank test.

### Mutational status

In 13 patients we failed to receive tumour tissue for mutational testing and consequently mutational status was assessed in a total of 94 patients. *KRAS *mutations were detected in 41 patients (44%), as presented in table 2. Only 3 patients had BRAF mutations and these were all *KRAS *wt tumours as show in table 3. A similar mutual exclusiveness was not revealed by the *PIK3CA *analysis, where 7, 5 and 1 patient harboured mutations located in codon E542, E545 and E1047, respectively. Concomitant *PIK3CA *and *KRAS *mutations were observed in 8 patients. Loss of PTEN as defined by less than 50% of tumour cells positive for PTEN immunostaining was detected in 13 of the 73 (18%) tumours available for PTEN staining.

**Table 3:** Distribution of the different mutations

*Patient*	*KRAS mutations*	*BRAF mutations*	*PIK3CA mutations*
1	12ALA	wt	Wt

2	12ALA	wt	Wt

3	12ALA	wt	Wt

4	12ALA	wt	Wt

5	12ARG	wt	Wt

6	12ARG	wt	Wt

7	12ARG	wt	Wt

8	12ASP	wt	E542K

9	12ASP	wt	E542K

10	12ASP	wt	E545K/D**

11	12ASP	wt	E545K/D

12	12ASP	wt	E545K/D

13	12ASP	wt	Wt

14	12ASP	wt	Wt

15	12ASP	wt	Wt

16	12ASP	wt	Wt

17	12ASP	wt	Wt

18	12ASP	wt	Wt

19	12ASP	wt	Wt

20	12ASP	wt	Wt

21	12ASP	wt	Wt

22	12ASP	wt	Wt

23	Mut*	NA	NA

24	12CYS	wt	E542K

25	12CYS	wt	Wt

26	12CYS	wt	Wt

27	12SER	wt	Wt

28	12SER	wt	Wt

29	12VAL	wt	E542K

30	12VAL	wt	H1047R

31	12VAL	wt	Wt

32	12VAL	wt	Wt

33	12VAL	wt	Wt

34	12VAL	wt	Wt

35	12VAL	wt	Wt

36	13ASP	wt	Wt

37	13ASP	wt	Wt

38	13ASP	wt	Wt

39	13ASP	wt	Wt

40	13ASP	wt	Wt

41	13CYS	wt	Wt

42	wt	E600	Wt

43	wt	E600	Wt

44	wt	E600	Wt

45	wt	wt	E542K

46	wt	wt	E542K

47	wt	wt	E542K

48	wt	wt	E545K/D

49	wt	wt	E545K/D

*KRAS mutational status assessed in external institution, but not specified.

**The DxS assay does not discriminate between the E545K and E545D mutation.

### Mutational profiling and clinico-pathologic characteristics

No significant association was revealed between *KRAS*, *BRAF*, *PIK3CA*/PTEN mutational status/loss of staining and age, gender, tumour location or any of the pre-treatment characteristics listed in table 1 (Data not shown).

### Mutations in KRAS, BRAF or/and PIK3CA/PTEN and clinical outcome

Clinical outcome and mutational status are presented in table 2 and revealed response rates between 20% and 37% in patients tested negative for either mutations.

All three patients with *BRAF *mutation were non responders and furthermore there was a lower DC rate, median PFS and OS in patients carrying this mutation. The statistical analysis on *BRAF *mutations did not reach significance, which was most probably due to the low number (3) of patients with *BRAF *mutations detected.

The *PIK3CA *mutational analysis revealed a significantly clinical benefit in terms of response, disease control, as well as PFS and overall survival in patients with *PIK3CA *wild type status. Only this mutation reached statistical significance in univariate overall survival analysis. Patients with *PIK3CA *mutations achieved a median PFS of 2.3 month and a median OS of 5.8 months compared to 4.6 and 9.2 months, respectively, in patients who did not carry these mutations.

Only one of the 13 patients with loss of PTEN (8%) achieved response compared to 29% of patients with normal PTEN expression. The difference was not significant, and even less pronounced with respect to disease control rates in the two groups. Similarly, there was no significant difference in PFS or OS between the two groups. Consequently, this marker was excluded for further analysis, and the *KRAS, BRAF *and *PIK3CA *mutations were selected for combined mutational analysis.

### Triple mutational status and clinical outcome

**Figure 1:**
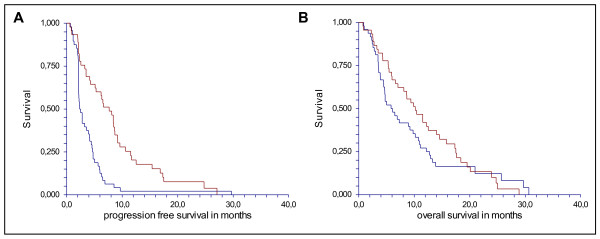
**Progression free survival (A) and overall survival (B) in triple negative patients and patients with any mutations detected**. The median progression free survival was significantly higher in patients with triple negative mutational status (Red: 7.7 months (5.1-8.6, 95%CI)) compared to patients with one or more mutations (Blue: 2.3 months (2.1-3.6, 95% CI) P < 0.000). The HR was 2.24 (1.45-3.47 95% CI). The median OS was significantly higher in patients with triple negative mutational status (Red: 10.2 months (7.1-12.5, 95%CI)) compared to patients with one or more mutations (Blue: 5.8 months (4.5-9.2, 95% CI) p = 0.31). The HR was 1.23 (0.81-1.89 95% CI).

All the 49 patients with *KRAS, BRAF *or/and *PIK3CA *mutations were non-responders, and consequently the response rate in patients with triple negative mutational test vas 42% p < 0.0001). Furthermore, these patients achieved a disease control rate of 73% (compared to 41% in the mutational group, p = 0.001) and the median progression free and overall survival rates were increased to 7.7 and 10.2 months, respectively in this group of patients. Notably, 22% of the patients with one or more mutations showed early progressed before the third cycle compared to 7% in the triple negative group (p = 0.03). Kaplan-Meier plots and univariate survival analysis are presented in figure 1.

## Discussion

*KRAS *mutations only accounts for 30-40% of the non-responders to EGFR targeted MoAbs in colorectal cancer. Considering the fact that these agents are now established treatment options in metastatic colorectal cancer and widely used, identification of genetic determinants of primary resistance in *KRAS *wild type tumour would be essential to further development. The present study investigated relevant mutations related to downstream signalling, which have shown promising results in a few studies.

The purpose of the EGFR analysis was to assess a possible predictive value in KRAS wild type patients only, since most previous data have failed to distinguish between KRAS wt and mutated patients. The EGFR expression by immunohistochemistry was evaluated in 68 patients and did not provide any additional predictive or prognostic value to mutational testing, as suggested by Yen et al [17]. Our findings are supported by the results from a phase III study which investigated chemotherapy and bevasizumab with or without cetuximab (Phase III CAIRO2) as first line treatment for mCRC. EGFR IHC was not a predictive marker in this setting of oxaliplatin based first line regime[19]. Thus, EGFR testing prior to treatment has limited utility, even when accounting for KRAS mutational status.

We confirm the previous literature on *KRAS *mutational analysis regarding association to non-responsiveness and PFS, but not OS. Of note, the present frequency of 44% mutated tumours is marginally higher than previously presented[3,4].

All *BRAF *mutant tumours were non-responders and all mutually exclusive from mutated KRAS. The overall *BRAF V600E *mutational rate was 3% compared to approximately 8-10% reported in the literature[2,4,7,12,20-23]. The low frequency could be a natural statistical variation (The 95% CIs of 3/100 are 0.6-8.5). Also, the frequency of *BRAF *mutations is expected to vary between different studies according to criteria for patient selection, line of treatment, PS, limited or advanced disease or further characteristics like geographic variation and microsatellite status amongst others. Furthermore, heterogeneity of *BRAF *mutational status in tumour compared to metastasis may complicate results. The low frequency of *BRAF *mutations may also rely on methodological aspects. The method used in our study was therefore validated with a sensitive in-house PCR method a well as direct sequencing, and the results were in complete agreement (data not shown). The *BRAF *analysis accounted for additional 6% of non-responders in this study, and is consequently regarded as clinical relevant for further selection, as confirmed by a recent pooled analysis of a larger sample size[4].

The PIK3/PTEN pathway is crucial to EGFR signalling and PTEN evaluation is very central to regulation[10]. Unfortunately the PTEN gene is large and mutations result in a loss of function and therefore not located in hotspots. Consequently, loss of PTEN protein evaluated by immunohistochemistry is a more attractive approach to mutational detection than a complete DNA sequencing. However, the value of PTEN IHC has been debated. In contrast to the studies by Perroni et al and Sartori Bianchi et al [12,13], two other studies failed to demonstrate a significant impact on response by the PTEN assessment[24,25]. We aimed to investigate the predictive and prognostic influence of this marker but found it limited in the present material. Another recent study suggested that PTEN IHC was a potential predictive marker when stained on metastatic but not on primary tissue as used in the present study[16]. Methodological difficulties in demonstrating a lack of expression by a semi quantitative method could be an obvious explanation for these findings. Sequencing of the PTEN gene may contribute further to the knowledge. It is however, a complicated and demanding method, and the analysis is probably not justified for use as a clinical tool.

The *PIK3CA *mutations were detected in 13% percent of available patients, which is in agreement with the previous literature[4,6,10,11,13,26]. *A *few publications have assessed the clinical impact of these mutations in EGFR targeted therapies. Perrone and colleagues described a significant impact on response and PFS by *PIK3CA/PTEN *mutations in a small study of 32 patients[12], and Sartori-Bianchi et al presented a significant PFS and OS survival benefit in non-mutated tumours, and suggested that these mutations may be a rational supplement to *KRAS *mutational testing with potential for clinical application[13]. However, these results are contradicted by a recent retrospectively study of 200 patients with mCRC, which revealed that 5 of 22 patients carrying *PIK3CA *mutations responded to treatment, and it was concluded that this mutations do not play a major role in primary resistance to EGFR MoAbs[26]. A substantial overlapping between the *KRAS *and *PIK3CA *mutations was revealed. Consequently, the *PIK3CA *analysis was not suggested as a single marker of response. The study was based on a non-homogeneous patients material treated according to 4 different clinical trials with either monotherapy cetuximab or combination therapy, and is therefore not comparable to our patient material. We detected a similarly substantial overlapping between the *KRAS *and *PIK3CA *mutations, but all *PIK3CA *mutated tumours in the present study were non-responders. A recent pooled analysis included data from 1022 patients with mCRC who were treated with EGFR Moabs (according to a variety of treatment regime and lines. The study investigated *KRAS, NRAS *and *BRAF *together with different *PIK3CA *mutations and has confirmed our result, but also suggested a significant different outcome according to the different type of *PIK3CA *mutations[4]. In the present study all patients with *PIK3CA *mutations were non-responders, and we were consequently unable to contribute to this aspect.

The sample size of the present study did not allow for comparison of methodological aspects regarding sensitivity for *BRAF/PIK3CA *mutations, or analysis of potential clinical differences according to the specific locations of the *KRAS *mutations[4,16]. These aspects will need assessment in larger studies. The same applies to development of assays for detection of less frequent *KRAS*, *PIK3CA *and *BRAF *mutations and the clinical implication of these. However, this study included a homogeneous group of patients who received identical treatment dose and schedule in a single institution, in comparison to the majority of recent studies. The observation period allowed for data collection on progression and survival, and revealed a large number of events (97) for survival analysis. Only four patients had not progressed at time of analysis and consequently the strength of statistical analysis on multiple parameters appears acceptable. The median progression free survival in triple negative patients was 7.7 months and held a median overall survival of 10.2 months, indicating a clear benefit from treatment in this group of patients compared to those with one or more mutations. Despite the present limitations, by adding the *BRAF *and *PIK3CA *mutational analysis *to KRAS *testing we identified additional 11% of the non-responders.

## Conclusion

In conclusion, it is a major challenge to identify patients with a considerable risk of unnecessary toxic side effects and limited benefit from treatment. The present data suggest that patients harbouring any of the three mutations have a very low chance of response and a high risk of early progression before the third cycle of treatment. Furthermore, the triple negative patients achieved a significantly higher disease control rate which translates into a marked increase of progression free survival rate compared to patients with any of the three mutations. Consequently, we suggest that patient selection for third-line combination therapy with EGFR monoclonal antibodies and irinotecan could be based on triple mutational testing.

## Competing interests

The authors declare that they have no competing interests.

## Authors' contributions

KGS was responsible for design, concept, protocol writing, implementation, patient inclusion and treatment, immunohistochemical scoring, clinical data collection, data analysis, writing of manuscript and approval.

NP implemented and analysed mutational data, participated in manuscript writing and approved of the final manuscript.

JL supervised immunohistochemistry, performed second scoring, participated in writing and approved the final manuscript.

SKF treated patients and participated in second scoring of immunohistochemistry, and approved the final manuscript.

AJ was responsible for patient treatment, study concept and design and participated in writing and final approval of the manuscript.

## Pre-publication history

The pre-publication history for this paper can be accessed here:

http://www.biomedcentral.com/1471-2407/11/107/prepub
